# 

**Published:** 1889-05

**Authors:** 


RECEIPTED TO JAN. 1890—J. R. Muse, C S Priestley, M W Speer, M P Deadwyler, J H Young, J E Pope, C F Yeager, W H Boykin, C. Bethune, J W Pinkston, W A Monroe, John Tyler, Ja T. Meredith, William Boag, Robt. L Smythe; J W Bentley, to Apr. '90; G B Wade, to Apr. '90; J A Stewart, to Jan. '89; W T Foute, to July, '89; J F Brooks, to Apr. '89; S R Mitchell, to July, '89; W J Westbrook, to Sep., '89; A S Miller, to Apr. '89; J T Kirkpatrick, to Jan., '91.

